# Addressing the Critical Gap in Biological Therapies for Inflammatory Bowel Disease in Syria

**DOI:** 10.1055/s-0045-1809039

**Published:** 2025-05-21

**Authors:** Marouf Mouhammad Alhalabi

**Affiliations:** 1 Department of Gastroenterology / Internal Medicine, Damascus Hospital, Damascus, Syria

Syria's evolving health care landscape demands urgent attention to the unmet therapeutic needs of patients with inflammatory bowel disease (IBD). The discontinuation of biological therapies—cornerstones of modern IBD management—has deprived patients of treatments proven to induce remission, promote mucosal healing, and reduce complications in Crohn's disease and ulcerative colitis. 1 2 Systemic governance failures and economic sanctions have severely restricted access to Food and Drug Administration (FDA)-approved biologics, leading to reliance on unapproved biocopies.

This practice raises significant safety concerns and ethical dilemmas, as patients are often unaware of the risks associated with untested alternatives. Historically, Syria's pharmaceutical infrastructure has been eroded by underinvestment, hospital destruction, and workforce attrition, reflecting a broader pattern of systemic neglect. 3 4

The clinical implications are profound. Patients now face heightened risks of disease flare-ups, hospitalizations, and surgical interventions. Substandard biocopies, which lack rigorous pharmacokinetic validation, correlate with inconsistent outcomes, increased morbidity, and potential harm. These challenges contravene principles of evidence-based medicine and patient autonomy, as individuals are deprived of informed consent regarding therapeutic risks. 5 6

Reinstating access to biologics demands robust multisectoral collaboration. Policymakers must prioritize integrating FDA-approved agents—adalimumab, infliximab, and ustekinumab—into national protocols. This requires comprehensive training programs for health care professionals to optimize IBD management, particularly in severe cases such as steroid-refractory pancolitis. Concurrently, partnerships with international organizations (e.g., the World Health Organization and United Nations High Commissioner for Refugees) could address supply chain barriers and secure funding for sustainable access. 7 8

Ultimately, Syria's health care reforms must prioritize equitable access to biologics within a chronic disease strategy. Addressing this gap will mitigate preventable morbidity, reduce long-term costs, and align care with global standards. We urge the international medical community to advocate for policy changes and resource mobilization, ensuring Syrian patients receive therapies that meet universal safety and efficacy benchmarks. Restoring trust in Syria's health care system hinges on sustained advocacy, cross-border collaboration, and an unwavering commitment to patient-centered care. 9
